# Marginal zone lymphoma expression of histidine‐rich glycoprotein correlates with improved survival

**DOI:** 10.1002/jha2.73

**Published:** 2020-08-06

**Authors:** Tor Persson Skare, Elin Sjöberg, Mattias Berglund, Ross O Smith, Francis P Roche, Cecilia Lindskog, Birgitta Sander, Ingrid Glimelius, Alex R Gholiha, Gunilla Enblad, Rose‐Marie Amini, Lena Claesson‐Welsh

**Affiliations:** ^1^ Department of Immunology Genetics and Pathology Science for Life and Beijer Laboratories and Unit of Experimental and Clinical Oncology Uppsala University Uppsala Sweden; ^2^ Dept of Laboratory Medicine Division of Pathology Karolinska Institutet and Karolinska University Hospital Stockholm Sweden

**Keywords:** alpha defensin 1, gene expression, histidine rich glycoprotein, marginal zone lymphoma, survival

## Abstract

**Purpose:**

The abundant hepatocyte‐expressed plasma protein histidine‐rich glycoprotein (HRG) enhances antitumor immunity by polarizing inflammatory and immune cells in several mouse models, however, the clinical relevance of HRG in human cancer is poorly explored. The expression and role of HRG in human B‐cell lymphomas was investigated in order to find new tools for prognosis and treatment.

**Findings:**

Immunohistochemical (IHC) analysis and RNA hybridization of tissue microarrays showed that (i) HRG was expressed by tumor cells in marginal zone lymphoma (MZL), in 36% of 59 cases. Expression was also detected in follicular lymphoma (22%), mantle cell lymphoma (19%), and indiffuse large B‐cell lymphoma (DLBCL;5%) while primary CNS lymphoma (PCNSL) lacked expression of HRG. (ii) MZL patients positive for HRG showed a superior overall survival outcome (HR = 0.086, 95% CI = 0.014‐0.518, *P*‐value = .007), indicating a protective role for HRG independent of stage, age and sex. (iii) HRG‐expressing MZL displayed significantly increased transcript and protein levels of the host defense peptide alpha defensin 1. In addition, global transcript analyses showed significant changes in gene ontology terms relating to immunity and inflammation, however, infiltration of immune and inflammatory cells detected by IHC was unaffected by HRG expression.

**Conclusion:**

HRG expression by MZL tumor cells correlates with an altered transcription profile and improved overall survival.

## 1 INTRODUCTION

Mature B‐cell lymphomas are the most common type of lymphoid neoplasms, constituting over 90% of lymphomas and approximately 4% of all new cancer cases [1]. Marginal zone lymphomas (MZL) arise in the marginal zone of B‐cell follicles and are subdivided into splenic (SMZL), nodal (NMZL), and extranodal MZL of mucosa‐associated lymphoid tissue (MALT) [2, 3]. MALT is the most common type of MZL constituting 5‐8% of all B‐cell lymphomas, while NMZL and SMZL constitute approximately 2% each [4]. The incidence of MALT increases with age and is particularly common in patients older than 80 years [5, 6]. Although there are common genetic and clinicopathological features among the MZL subtypes, both prognosis and treatment protocols differ between the subtypes. Most SMZL patients have a slow disease progression, and the median patient survival is 8‐10 years. However, SMZL has a heterogeneous clinical course with approximately 25% of the patients surviving only 3‐4 years and 5‐10% of patients undergoing transformation to diffuse large B‐cell lymphoma (DLBCL) [7]. Studies are therefore warranted to identify patients who will present with an aggressive disease course and benefit from chemotherapy. Also in other indolent B‐cell lymphomas, such as follicular lymphoma (FL) and mantle cell lymphoma (MCL), the disease course is variable and tools to identify different prognostic subgroups are urgently needed [8].

In the healthy individual, histidine‐rich glycoprotein (HRG; 75 kDa) is exclusively produced by hepatocytes and is present in plasma at a concentration of 100‐150 ug/mL [9]. HRG consists of two N‐terminal cystatin‐like domains, followed by a histidine/proline‐rich domain organized in 12 pentapeptide repeats. HRG binds divalent metal ions including Zn^2+^ as well as heparan sulfate/heparin and heme through its histidine‐rich region [10]. In several mouse tumor models HRG exerts gene regulatory effects in tumor‐associated macrophages resulting in a switch from an M2 to M1 polarity profile, correlating with enhanced antitumor immunity, decreased tumor growth, and reduced metastatic spread [11]. Treatment of mouse glioma by HRG‐adenovirus gene therapy results in reduced infiltration of regulatory T cells and suppressed tumor growth [12]. In humans, HRG serum levels are increased in breast cancer patients as compared with healthy controls [13]. In contrast, in ovarian cancer, HRG serum levels decrease in advanced cancer, compared to lower stage cancer and healthy controls [14]. Whether this decrease is due to impaired liver function, or direct effects on HRG transcript and protein production/turnover remain to be addressed. In sepsis, HRG plasma levels decrease and therefore, monitoring plasma HRG can serve as a sepsis biomarker [15].

The immune modulatory effects of HRG prompted the question of a potential involvement of HRG in lymphoma progression. Here, we show that HRG is expressed in several mature B‐cell lymphomas. In MZL, HRG is produced by the tumor cells and the expression correlates with an altered transcription profile and with improved overall survival.

## 2 MATERIALS AND METHODS

### 2.1 Patient material

Patients were diagnosed at the Department of Pathology, Uppsala University Hospital, which is a referral center for lymphoma diagnostics in the Uppsala Health Care region, and from the Department of Pathology, Karolinska University Hospital. MZL (n = 59) were diagnosed between 1993 and 2016, follicular lymphomas (FL) (n = 51) between 2005 and 2015, mantle cell lymphomas (MCL) (n = 31), DLBCL (n = 139) between 2002 and 2016, and primary DLBCL of the CNS (PCNSL) (n = 33) during 1993‐2014. All diagnoses were re‐evaluated by an experienced hematopathologist before the construction of tissue microarrays (TMAs). Representative areas of lymphoma tissue to be included in the TMA were selected containing >70% of tumor cells, and each patient was represented by two 1 mm TMA cores. The majority of splenic MZL patients were treated with splenectomy, while nodal MZL patients were, if localized, treated with 30 Gy radiotherapy or, if generalized disease, treated with single rituximab, or rituximab in combination with chemotherapy; chlorambucil, CHOP (cyclophospamide, doxorubicin, vincristine, and prednisone), or bendamustine. Prior to treatment, *Helicobacter pylori* infection was eradicated. All clinical characteristics were collected from medical records. The study was approved by the Regional Ethical Committee in Uppsala (Dnr 233/2014, 2012/127).

### 2.2 Immunohistochemical staining

Slides were stained for HRG using a standard immunohistochemical (IHC) protocol from the Human Protein Atlas (https://www.proteinatlas.org). In brief, tissue slides were deparaffinized and hydrated in xylene and graded ethanol, respectively. Endogenous peroxidases were blocked in 0.3% hydrogen peroxide in 95% ethanol, for 5 min. For antigen retrieval, slides were boiled in retrieval buffer, pH 6, at 125°C for 4 min in a pressure boiler (Decloaking chamber, Biocare Medical, Walnut Creek, CA, USA) followed by cooling to 90°C, in total a protocol of ∼40 min. Automated IHC staining was performed using Autostainer 480 (ThermoFisher Scientific, Waltham, MA, USA) and Autostainer XL (Leica Biosystems, Vista, CA, USA). Slides were blocked with Ultra V Block for 5 min, followed by washing and incubation with primary antibodies recognizing HRG produced in‐house, HRG‐0119 for 30 min [16]. Next, slides were rinsed in wash buffer and incubated for 30 min with UltraVision LP HRP Polymer visualization probe (ThermoFisher Scientific) for 30 min and developed with Diaminobenzidine (ThermoFisher Scientific) for 10 min. Slides were then counterstained with Mayer's hematoxylin (Histolab Products AB, Göteborg, Sweden) and mounted with Pertex mounting medium.

Automated IHC stainings for CD3, CD4, CD8, CD20, and CD68 were performed using Dako Autostainer Link 48 with Dako Envision FLEX high pH Detection Kit (#K8000) with the following antibodies, all from Dako: CD3 (GA503 polyclonal, RTU), CD4 (#IR649, mouse monoclonal, clone 4B12, RTU), CD8 (#IR623, mouse monoclonal, clone C8/144B, RTU), CD20 (#IR604/GA604, clone L26, RTU), and CD68 (#IR613, mouse monoclonal, clone PG‐M1).

### 2.3 RNA in situ hybridization

In situ hybridization was performed on the MZL TMAs using the RNAscope 2.5 HD Red assay (catalog no. 322350; Advanced Cell Diagnostics, Newark, CA, USA). A target probe for HRG (NM_000412.4) and negative control probe for DapB (bacterial dihydrodipicolinate reductase) were used. Slides were de‐paraffinized with xylene and rehydrated with 100% ethanol followed by hydrogen peroxide treatment. Next, slides were boiled for 20 min in target retrieval solution and permeabilized with protease plus, for 30 min. After permeabilization, slides were hybridized with the probes for 2 h at 40°C followed by signal amplification steps and counterstaining with Mayer's hematoxylin (Histolab Products AB, Göteborg, Sweden).

### 2.4 Immunofluorescent multiplex staining

Slides were double‐stained for Marginal zone B and B1 cell‐specific protein (MZB1; HPA043745, Atlas Antibodies AB, diluted 1:2000) and Alpha defensin 1 (DEFA; HPA052517, Atlas Antibodies AB, diluted 1:600) and nuclei were visualized with DAPI. Deparaffinization, antigen retrieval, blocking, incubation with primary antibodies, secondary HRP polymer, and washing steps were performed in the same manner as for immunohistochemistry, described above, with the addition of two additional washing steps. For visualization, slides were incubated with Tyramide Signal Amplification (TSA) Substrate (TSA‐Plus, PerkinElmer, MA, USA) for 15 min. Each antibody was added in a separate staining cycle and visualized with different TSA fluorophores; Cyanine 3 (MZB1, yellow) and Cyanine 5 (DEFA1, red), diluted in amplification diluent (PerkinElmer). Between the cycles, slides were boiled in retrieval buffer at 90°C degrees for 20 min for elution of the first primary antibody. Finally, slides were mounted with Fluoroshield containing DAPI (Abcam, Cambridge, UK).

### 2.5 Gene microarray analysis

RNA was isolated from paraffin‐embedded slides using RNeasy FFPE kit (Qiagen). RNA concentration was measured by NanoDrop (Thermo Fisher Scientific), and RNA integrity number (RIN) was assessed by Bioanalyzer (Agilent). Microarray assay was performed using Clariom™ DPico Assays, Human (Thermo Fisher Scientific) with RNA from five HRG‐negative and four HRG‐positive patient samples. The data were deposited at the Gene Expression Omnibus (GEO) database repository; accession number: GSE151360.

### 2.6 Image acquisition and analysis

TMAs used for IHC staining or RNAscope hybridization were scanned at 20× using an Aperio Scanscope CS slide scanner system and analyzed with Aperio ImageScope software (Aperio technologies, Vista, CA, USA). Scoring was performed using a Nikon Eclipse 80i microscope at 20×. Patients were scored either negative or positive for HRG protein expression and RNA Scope signal. Patients with the presence of HRG‐positive tumor cells in a TMA core were scored positive. All HRG‐positive patients had >10 HRG‐positive tumor cells/core. Scoring for DEFA1 and MZB was similarly performed. Scoring was performed independently by two researchers and re‐evaluated for the limited number of cases where the scoring differed between them. Scoring for the immune markers CD3, CD4, CD8, and CD68 were done by dichotomizing expression as either high or low relative to the other cores on the same TMA. Scoring for immune cells was performed by a researcher together with a pathologist. Researchers were blinded for clinical characteristics before scoring.

### 2.7 Statistical analysis

Pearson Chi‐square test was used for analyzing association of HRG RNA and protein expression, and HRG levels with the immune cell markers CD3, CD4, CD8, and CD68. The Kaplan‐Meier method and log‐rank test were used to estimate overall survival and Cox proportional hazards regression model was used for comparing hazard rates in univariable and multivariable analyses. Multivariable analyses were adjusted for age (dichotomized over/under 65), sex, and stage. Stage was dichotomized to either high or low stage where Ann Arbour stage < 2 or Musshoff < Pe II were categorized as low stage. The SPSS software package 21.0 (IBM Corporation, Armonk, NY, USA) was used for statistical analysis and *P‐*values < .05 were considered statistically significant. Differential gene expression from the gene microarrays was assessed using the linear model with coefficient evaluation by moderated *t*‐test. R software version 3.5.1 (R Foundation for Statistical Computing, Vienna, Austria) and Limma package version 3.38.3 were used for the analysis [17].

## 3 RESULTS

### 3.1 HRG protein expression in mature B‐cell lymphomas

**FIGURE 1:**
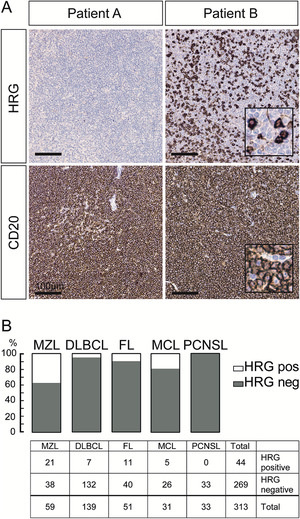
HRG protein expression in mature B‐cell lymphomas. A, Representative images of positive (Patient B) and negative (Patient A) HRG IHC staining patterns in marginal zone lymphoma (MZL) tissue micro arrays (TMAs). Both positive and negative samples showed abundant CD20‐positive tumor cells. Insets in patient B images show magnification of typical HRG‐positive and CD20‐positive tumor cells. B, Proportion and total number of patients with positive and negative HRG expression in MZL, diffuse large B‐cell lymphoma (DLBCL), follicular lymphoma (FL), mantle cell lymphoma (MCL), and primary CNS lymphoma (PCNSL) TMAs. Scale bar in (A)100 μm

Although there is ample evidence from mouse models that HRG's impact on inflammation and antitumor immunity can steer tumor progression, HRG's clinical relevance and the molecular mechanisms for its potential role in human cancer remain to be fully explored. We therefore examined the expression and potential role for HRG in human lymphomas. Mature B‐cell lymphoma TMAs including MZL (n = 59, see Table I), FL (n = 51), MCL (n = 31), DLBCL (n = 139), and PCNSL (n = 33) were immunostained for the presence of HRG in the tumor compartment (Figure S1). HRG‐positive tumor cells, identified based on morphology (see inset in Figure 1A), were present in patient subsets. In MZL, 36% of the samples showed positive staining for HRG, while for the other diagnoses, fewer cases were positive; 22% of FL, 19% of MCL, and 5% of DLBCL cases were positive for HRG to variable degrees between samples (Figure 1B). In contrast, all samples from PCNSL were negative for HRG (Figure 1B). Parallel immunostaining for the B‐cell marker CD20 showed abundant CD20‐positive tumor cells in the biopsies, independent of the HRG score (Figure 1A, lower panel). In conclusion, HRG protein is present in several mature B‐cell lymphoma types with MZL having the highest proportion of HRG‐positive patients.

**TABLE 1:** MZL patient cohort characteristics

	HRG^+^	HRG^‐^
**SEX**		
MALE	8	10
FEMALE	13	28
**AGE**		
MEAN	65.2	66.1
OVER 65	9	21
UNDER 65	12	17
**MZL SUBTYPE**		
SMZL	14	18
NMZL	0	3
MALT	5	11
MISSING	3	5
STAGE*		
HIGH	12	22
LOW	8	12
MISSING	2	3
**5‐YEAR SURVIVAL**	87%	62%

* Lymphoma stage were dichotomized into high and low, with Ann Arbour ≤II or Mushoff < Pe II defined as low and > II as high.

### 3.2 HRG is expressed by lymphoma tumor cells

**FIGURE 2:**
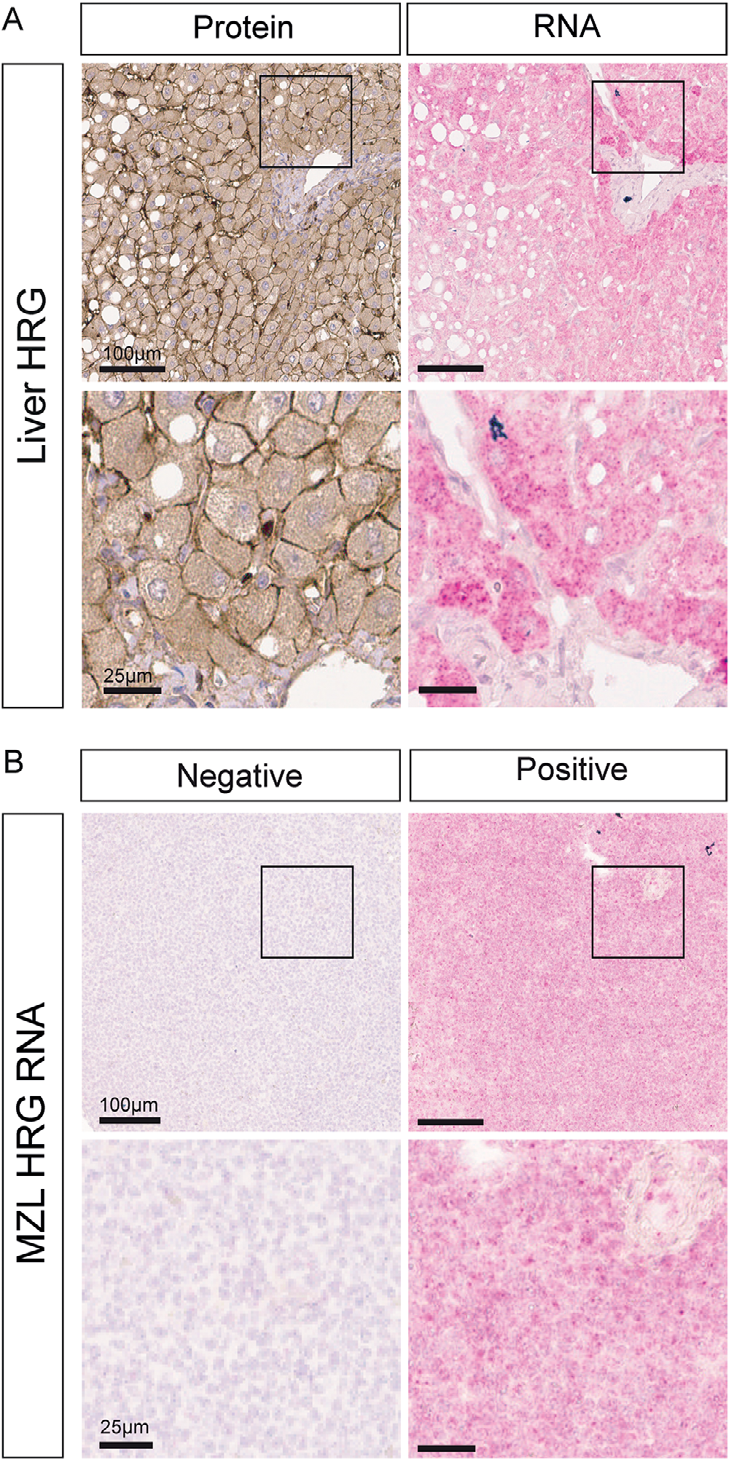
HRG mRNA is expressed in healthy liver tissue and MZL. A, Top: HRG mRNA and protein expression in consecutive sections of healthy liver tissue. Scale bar, 100 μm. Bottom: magnification of box in upper row. Scale bar, 25 μm. B, Top: Representative images showing presence or lack of HRG transcripts in MZL samples. Scale bar, 100 μm. Bottom: magnification of box in upper row. Scale bar, 25 μm

HRG is predominantly produced by liver hepatocytes, distributed throughout the circulation, and deposited in the perivascular stroma in healthy tissues [11]. To determine if HRG protein detected in lymphomas is produced in the liver or expressed locally by lymphoma tumor cells, the presence of HRG transcripts in the tumor samples was investigated using RNAscope. First, healthy liver tissue was used to evaluate the accuracy of the HRG RNAscope probe in parallel with antibody staining on consecutive tissue sections (Figure 2A). As expected, HRG mRNA and protein were highly expressed by hepatocytes, thereby validating the RNAscope probe. Next, RNA‐scope was performed on a subset of the MZL samples (Figure 2B). Expression of HRG mRNA was found in 8 of 19 samples and HRG mRNA was significantly associated with HRG protein expression (*P*‐value = .04; Table S1).

These data show that although the HRG protein found in 36% of the MZL cases may be produced in the liver and brought to the tumor by the circulation, it is also endogenously produced by the cancer cells in the lymphoma biopsies.

### 3.3 HRG is an independent marker for improved overall survival in MZL

**FIGURE 3:**
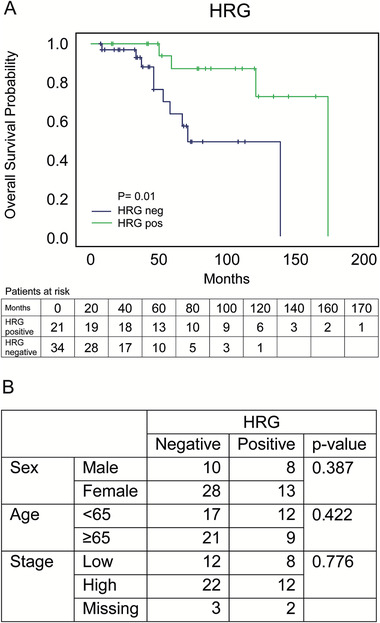
HRG‐positive MZL patients have better overall survival. A, Kaplan‐Meier analysis of overall survival in MZL patients for HRG expression. Statistical analysis using log‐rank test, *P* = .01. B, Association table between HRG status and sex, age and lymphoma stage. Low stage is defined as Ann Arbor II or lower or Musshoff Pe II or lower. Statistical analysis with Pearson Chi‐Square

To a variable degree, we found HRG expression in MZL, FL, MCL, and DLBCL samples. The relatively low prevalence in FL and DLBCL combined with the small patient cohort in MCL made it infeasible to further evaluate these diagnoses. We therefore focused on MZL for evaluation of the clinical implication of HRG‐positive tumor cells, and assessed the HRG protein score in relation to patient survival data. Of the 59 MZL patients, survival data were available for 55 patients. Kaplan‐Meier analysis demonstrated a significantly better overall survival of patients scored positive for HRG (*P*‐value = .01, log‐rank test; Figure 3). Univariable Cox‐regression analysis showed a decreased hazard rate for patients positive for HRG (HR = 0.188, 95% CI = 0.049‐0.721, *P*‐value = .015, n = 55). HRG status was not associated with differences in age, sex, or stage (Figure 3), and a multivariable analysis including these parameters showed that HRG‐status independently predicts improved overall survival (HR = 0.086, 95% CI = 0.014‐0.518, *P*‐value = .007, n = 54; Table S2). These data show that HRG in a subset of cancer cells in the tumor is of clinical relevance for MZL patient prognosis.

### 3.4 HRG expression does not correlate with inflammatory/immune cell status but is associated with increased expression of alpha defensin1

To characterize the inflammatory/immune cell status, IHC stainings were performed on the MZL samples against CD3 (T cell lineage), CD4 (T helper cells), CD8 (T killer cells), and CD68 (monocytic lineage). MLZ samples were scored as either low or high for these markers and the scores were compared to the presence of HRG‐positive cells. Results showed no significant association between the presence of HRG and infiltration of macrophages or T‐lymphocytes (Table S3). However, a subset of HRG‐positive cells in the MZL patient samples showed a macrophage‐like morphology (Figure S2), supporting previous reports showing that HRG binds to the cell surface of monocytes [18].

**FIGURE 4:**
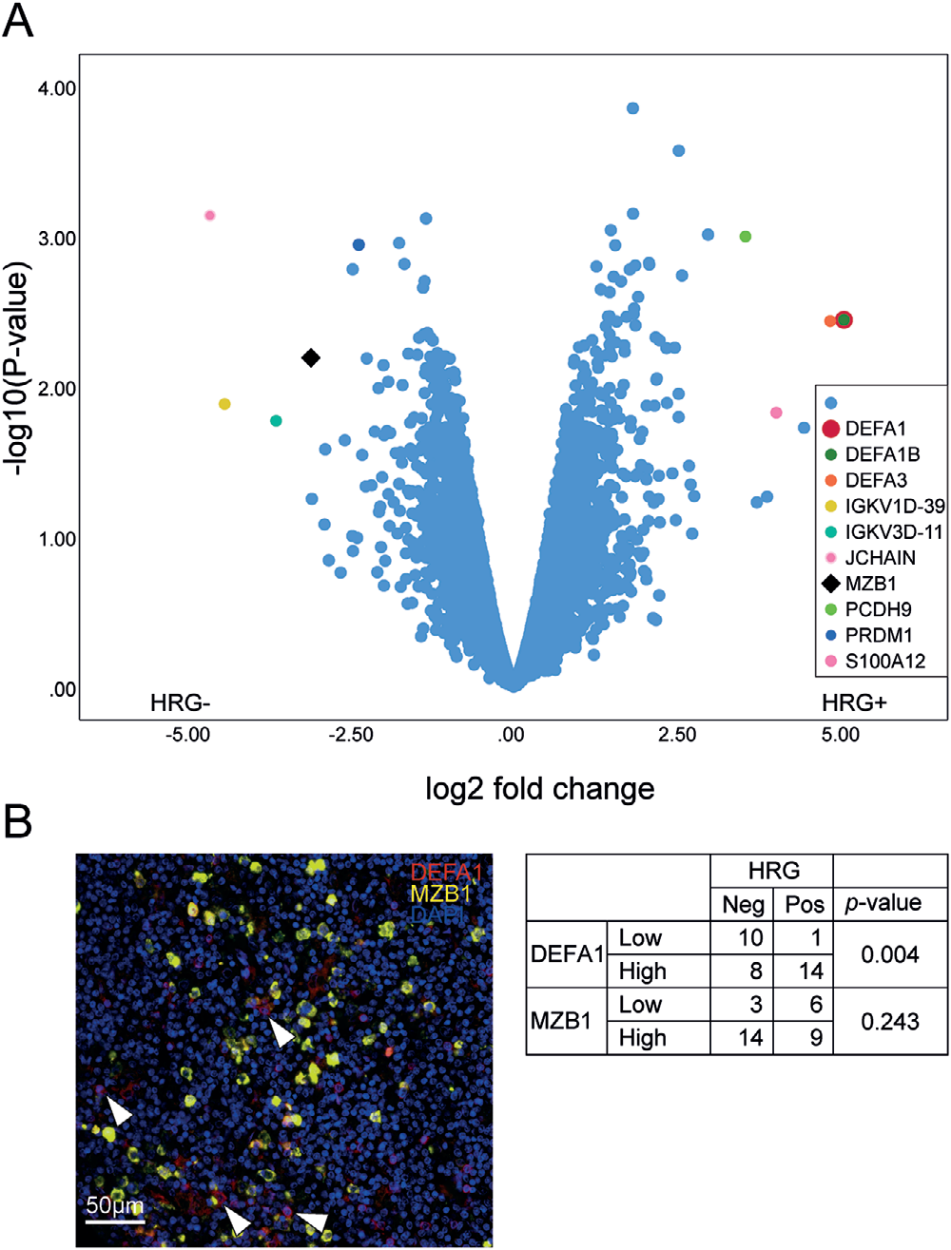
HRG‐positive patients have higher expression of alpha defensin 1 (DEFA1). A, Volcano plot showing results of transcript microarrays. Highlighted genes have log_2_ fold change > ±3 and *P*‐value < .05. B, Left: Immunofluorescence staining of DEFA1 (red), MZB1 (yellow), and DAPI (blue). Arrowheads indicate DEFA1 positive cells. Scale bar 50 μm. Right: Association table showing HRG status in relation to DEFA1 and MZB1. Note that scoring for DEFA1 positive cells may be an underestimation as DEFA1 is a secreted peptide. Statistical analysis with Fisher's Exact Test

To further explore the role of HRG expression in MZL, we performed gene expression microarray analyses. RNA was extracted from FFPE‐samples (four HRG‐positive and five HRG‐negative samples) followed by probing on Clariom D picoarrays. Genes that differed significantly between the two groups (cut‐off; log_2_‐fold difference, *P *≤ .05, n = 567) were clustered using the gene set enrichment analysis tool g:profiler (https://biit.cs.ut.ee/gprofiler/gost) resulting in 35 gene ontology (GO) terms (Table 2). A majority of the GO terms were related to immunity and inflammatory processes, indicating that these differed significantly between tumors expressing HRG or not. Genes with log_2_ fold change > ±3 and *P*‐value < .05 were highlighted in a volcano plot (Figure 4). The most markedly affected gene, alpha defensin 1 (*DEFA1*), present in HRG‐positive samples, and marginal zone B and B1 cell‐specific protein (*MZB1*), present in HRG‐negative samples were further examined using immunofluorescence staining. Patients were scored with regard to expression of MZB1 and DEFA1 and association analyses for HRG status were performed. While expression of MZB1 assessed by immunofluorescent staining failed to correlate with HRG status, there was a significant positive association between expression of HRG and DEFA1 (Figure 4).

**TABLE 2:** Gene ontology terms of differentially expressed genes in HRG‐positive and ‐negative MZL patients

Upregulated in HRG+ vs HRG‐ (303 genes)		
Gene ontology term		*P‐*value
Myeloid leukocyte activation	GO:0002274	2.52 × 10^‐07^
Leukocyte activation	GO:0045321	2.53 × 10^‐07^
Leukocyte activation involved in immune response	GO:0002366	3.39 × 10^‐07^
Cell activation involved in immune response	GO:0002263	3.93 × 10^‐07^
Leukocyte degranulation	GO:0043299	7.19 × 10^‐07^
Granulocyte activation	GO:0036230	1.08 × 10^‐06^
Myeloid cell activation involved in immune response	GO:0002275	1.10 × 10^‐06^
Myeloid leukocyte mediated immunity	GO:0002444	1.46 × 10^‐06^
Neutrophil degranulation	GO:0043312	2.10 × 10^‐06^
Neutrophil activation involved in immune response	GO:0002283	2.40 × 10^‐06^
Neutrophil activation	GO:0042119	3.84 × 10^‐06^
Neutrophil mediated immunity	GO:0002446	3.84 × 10^‐06^
Immune effector process	GO:0002252	4.88 × 10^‐06^
Cell activation	GO:0001775	1.11 × 10^‐05^
Cytokine production	GO:0001816	2.61 × 10^‐05^
Leukocyte mediated immunity	GO:0002443	8.67 × 10^‐05^
Regulated exocytosis	GO:0045055	9.24 × 10^‐05^
Vesicle‐mediated transport	GO:0016192	1.67 × 10^‐04^
Secretion	GO:0046903	4.63 × 10^‐04^
Regulation of cytokine production	GO:0001817	4.90 × 10^‐04^
Exocytosis	GO:0006887	5.68 × 10^‐04^
Immune response	GO:0006955	6.77 × 10^‐04^
Response to fungus	GO:0009620	9.96 × 10^‐04^
Secretion by cell	GO:0032940	1.68 × 10^‐03^
Cellular response to biotic stimulus	GO:0071216	2.33 × 10^‐03^
Export from cell	GO:0140352	3.85 × 10^‐03^
Innate immune response	GO:0045087	1.62 × 10^‐02^
Cellular response to molecule of bacterial origin	GO:0071219	1.97 × 10^‐02^
Immune system process	GO:0002376	2.00 × 10^‐02^
Positive regulation of cytokine production	GO:0001819	2.86 × 10^‐02^
Defense response to other organism	GO:0098542	3.78 × 10^‐02^
Response to external stimulus	GO:0009605	4.20 × 10^‐02^
Defense response	GO:0006952	4.75 × 10^‐02^
Upregulated in HRG‐ vs HRG+ (264 genes)		
immunoglobulin production	GO:0002377	3.54 × 10^‐06^
production of molecular mediator of immune response	GO:0002440	6.73 × 10^‐04^
adaptive immune response	GO:0002250	1.99 × 10^‐03^
immune system process	GO:0002376	7.82 × 10^‐03^
response to endoplasmic reticulum stress	GO:0034976	1.30 × 10^‐02^
protein N‐linked glycosylation	GO:0006487	2.50 × 10^‐02^

## 4 DISCUSSION

Here, we present for the first time evidence that HRG is expressed by tumor cells in MZL and is present to variable degrees in several other human mature B‐cell lymphoma types including FL, MCL, and DLBCL. A majority of the MZL patients in this study are diagnosed as SMZL and have undergone splenectomy. SMZL is an uncommon lymphoma type (<2% of all lymphomas) with a variable clinical course. After the introduction of systemic anti‐CD20 therapy (Rituximab), therapeutic splenectomy is no longer routine, which makes the patient material used here quite unique. The discovery of the significant increase in overall survival with HRG and DEF1A expression in SMZL, demonstrates the value in assessing gene and protein expression to tailor therapy regiments.

In MZL, HRG expression in tumor cells was detected in more than a third of samples, while the prevalence in the other lymphoma cohorts analyzed was lower. Indolent, low‐grade lymphomas like MZL and FL more frequently expressed HRG, whereas high‐grade lymphomas like DLBCL and PCNSL showed low or no expression. Of the B‐cell lymphomas, MCL presents with the most heterogeneous clinical pattern with some cases appearing as low‐grade lymphomas with an indolent clinical behavior, whereas others have an aggressive clinical course. The only cases of MCL with HRG expression were those primarily diagnosed in the bone‐marrow, with a leukemic profile. These particular tumors may belong to the group with a more indolent clinical behavior, although the number of cases was low [19]. These findings prompt future studies to explore whether the HRG‐positive patients in the different diagnoses of B‐cell lymphomas belong to a specific subgroup with common features. However, such analyses would require larger, well‐annotated cohorts, which currently are unavailable.

For indolent B‐cell lymphoma patients, the clinical course is very variable; a wait‐and‐watch approach is often considered for patients with slow progressive disease but biomarkers to identify patient groups that eventually need treatment are urgently needed [8]. Since the median age at diagnosis is usually high, many patients also suffer from comorbidities. A tailored treatment approach to minimize aggressive therapy for indolent cases is therefore important [20]. Thus, development of molecular tools for efficient therapy of B‐cell lymphomas is of high priority.

MZL patients with HRG‐expressing tumor cells showed a significantly improved overall survival as compared to HRG‐negative patients. Whether HRG expression is of clinical relevance in DLBCL, MCL, and FL needs to be established in future studies using larger patient material. The presence of HRG in cancer patient serum has previously been investigated as a prognostic biomarker in pancreatic adenocarcinoma, and in cancer of ovarian and breast cancer [13, 14, 21, 22]. In addition, HRG transcripts have been detected in the tumor microenvironment of breast cancer [23]. In tumor tissue from the cancer genome atlas (TCGA), high expression of HRG is seen in liver cancer as well as in rare cases of lung cancer and renal cancer [24]. The novel finding of HRG‐expression by lymphoma tumor cells prompts future studies to explore the expression of tumor cell‐derived HRG in other tumor types, and the correlation to clinicopathological characteristics and outcome.

Based on RNA expression data from the Human Protein Atlas, FANTOM5, and GTEx consortium, HRG is expressed almost exclusively in liver tissue in the healthy individual [25, 26, 27]. Hepatocyte‐derived HRG can regulate gene transcription in inflammatory cells, polarizing these cells toward an antitumor immunity phenotype secreting CD8^+^ T‐cell attracting cytokines [11]. In the MZL tumor samples in contrast, HRG expression did not correlate with an increased presence of CD4^+^ or CD8^+^ T cells, or CD68^+^ macrophages (Table S2). However, analyses of the gene expression micro arrays revealed activation of GO pathways involved in immune cell activation and immunity. Therefore, the improved survival of HRG‐expressing MZL patients might instead depend on infiltration of a specific subset of immune cells or through polarity effects on immune cells already present in the tumor tissue, resulting in enhanced antitumoral capacity. Another possibility is that early stages of HRG expressing MZL may display immune cell infiltration that becomes suppressed at later stages, but confirmation would require analysis of longitudinally collected samples and preferably, flow activated cell sorting to find rare cells. The scarcity of patient material however, makes such studies infeasible.

Distinct gene expression profiles were identified dependent on HRG expression. Of potential interest was the elevated levels of the B cell regulatory gene MZB1 in the HRG‐negative samples. MZB1 has been associated with adverse prognosis in leukemias and lymphomas [28]. We failed however to validate the expression of MZB1 by immunostaining. Another gene, the antimicrobial peptide alpha defensin 1, DEFA1, was significantly increased in HRG‐positive MZL patients, which was validated on the protein level by immunofluorescent staining. DEFA1 is produced by neutrophil precursors, and is stored in azurophilic granules of mature neutrophils [29]. It is also expressed at low levels by cancer cells in certain cancer forms including colorectal cancer and renal cell carcinoma [30]. Both HRG and DEFA1 have chemoattractant properties, and both can enhance the antitumoral effects of immune cells and increase macrophage phagocytosis [11, 29, 31, 32]. Thus, HRG expression by MZL tumor cells is accompanied by changes in gene expression, including *DEFA1*, characteristic of an antitumoral response. Future studies remain to establish whether gain of HRG expression is an early or late event in disease progression, and if the HRG‐associated GO profile, including *DEFA1*, is a cause or consequence of HRG expression. Finally, it remains to be shown whether HRG can be exploited not only as a diagnostic tool but also in therapy design.

## AUTHOR CONTRIBUTIONS

The study was instigated by GL, LCW, and RMA. TPS, ES, MB, ARG and FPR performed experiments.TPS, ES, MB and RMA scored tumor microarrays; TPS and ROS analyzed transcript microarrays. All authors analyzed and interpreted data. CL, BS, and IG provided clinical samples and clinical information. TPS, ES, RMA, and LCW wrote the manuscript and assembled figures; all authors commented on and agreed on the presentation. Main funding for the experimental part of the study was provided by LCW.

## CONFLICT OF INTEREST

The authors declare no competing financial interests for the current investigation.

5

## Supporting information

Supporting Information

## Data Availability

The data that support the findings in this study are available from the authors upon reasonable request. The array data have been deposited (see Methods)
