# Supplementary material for: Marginal zone lymphoma expression of histidine‐rich glycoprotein correlates with improved survival
Source: EJHaem. 2020 Aug 6;1(1):199–207. doi: 10.1002/jha2.73 (PMC9175683; doi:10.1002/jha2.73)
Supplement: Supplementary file 1 — Supporting Information [file JHA2-1-199-s001.pdf]

SUPPLEMENTAL MATERIAL

Marginal zone lymphoma expression of histidine-rich glycoprotein correlates with improved survival

Persson Skare T[1], Sjöberg E[1], Berglund M[1], Smith RO[1], Roche FP[1], Lindskog C[1], Sander B[2], Glimelius I[1], Gholiha A[1], Enblad G[1], Amini RM[1]* and Claesson-Welsh L[1]*

[1]Uppsala University, Department of Immunology, Genetics and Pathology, Science for Life and Beijer Laboratories, and Unit of Experimental and Clinical Oncology.

[2]Dept of Laboratory Medicine, Division of Pathology, Karolinska Institutet and Karolinska University Hospital, Stockholm

This file contains Supplemental figures 1 and 2 and Supplemental Tables 1-3.

Supplemental Figure 1

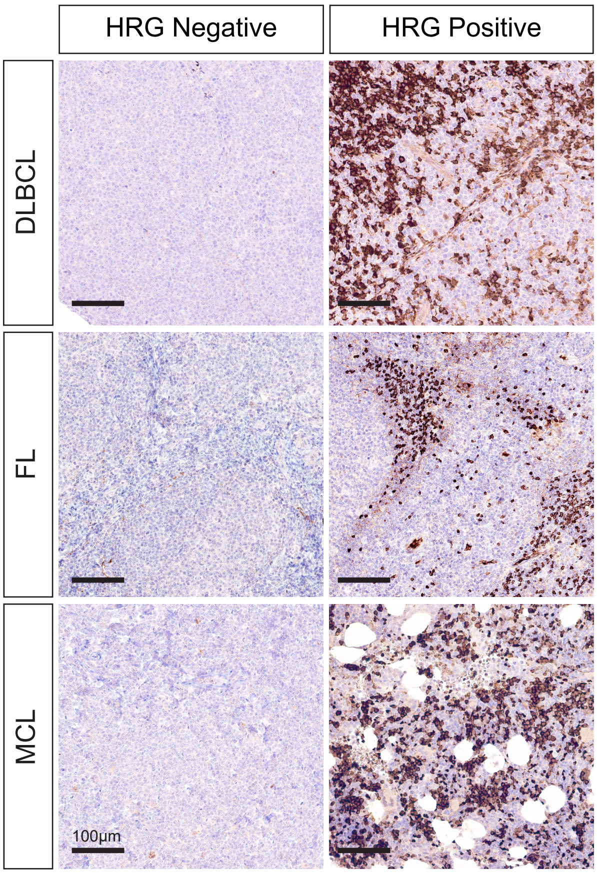

Supplemental figure 1. HRG is expressed by a subset of patients in several mature B-cell lymphomas. Representative images of positive and negative IHC stainings for HRG in DLBCL, FL and MCL patients. Scale bar 100 µm

Supplemental Figure 2

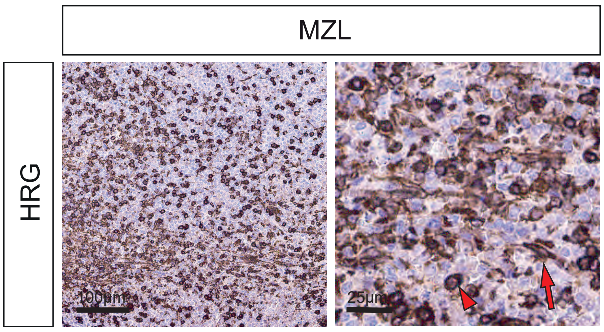

Supplemental Figure 2. HRG is expressed both in tumor cells and in macrophage-like cells. Left: HRG IHC staining of MZL patient sample. Right: Magnification of the image to the left. Arrowhead indicates HRG-positive lymphoma cell. Arrow indicate HRG-positive cell with macrophage-like morphology. Scale bar left 100 µm, right 25 µm.

Supplemental Table I. Co-expression of HRG protein and RNA in MZL

HRG expression In MZL		RNA*		
		Negative	Positive	Total
Protein**	Negative	8	2	10
	Positive	3	6	9
	Total	11	8	19

*) Detection by RNAscope
**) Detection by IHC

Supplemental Table II. Multivariate analysis for overall survival

		HR	CI	*p*-value
Age	<65	1		
	≥65	3.143	0.946-10.442	0.062
Sex	Male	1		
	Female	1.272	0.317-5.110	0.734
Stage	Low	1		
	High	17.134	1.715-171.147	0.016
HRG	Negative	1		
	Positive	0.086	0.014-0.518	0.007

Supplemental Table III. HRG expression and infiltrating cells of monocyte/macrophage and T cell lineages in MZL

		HRG		*p*-value
		Neg	Pos	
CD4	Low	7	5	
	High	16	11	0.614
CD3	Low	3	5	
	High	20	11	0.163
CD68	Low	12	5	
	High	11	11	0.167
CD8	Low	8	7	
	High	15	9	0.407

*Scoring showed no significant co-distribution of HRG expression and presence of infiltrating monocytes/macrophages and T cells in the MZL TMAs. $p>0.05$ (i.e. all obtained values in these analysis) was considered not significant.
